# Combining the Benefits of Homogeneous and Heterogeneous Catalysis with Tunable Solvents and Nearcritical Water

**DOI:** 10.3390/molecules15118400

**Published:** 2010-11-16

**Authors:** Ali Z. Fadhel, Pamela Pollet, Charles L. Liotta, Charles A. Eckert

**Affiliations:** 1 School of Chemical & Biomolecular Engineering, Georgia Institute of Technology, 311 Ferst Drive, Atlanta, GA 30332, USA; E-Mail: ali.fadhel@chbe.gatech.edu (A.Z.F.); 2 Specialty Separations Center, Georgia Institute of Technology, 311 Ferst Drive, Atlanta, GA 30332, USA; 3 School of Chemistry & Biochemistry, Georgia Institute of Technology, 901 Atlantic Drive, Atlanta, GA 30332, USA; E-Mail: pamela.pollet@chemistry.gatech.edu (P.P.)

**Keywords:** tunable solvent, nearcritical water, pressure induced heterogeneous separations, homogeneous catalysis

## Abstract

The greatest advantage of heterogeneous catalysis is the ease of separation, while the disadvantages are often limited activity and selectivity. We report solvents that use tunable phase behavior to achieve homogeneous catalysis with ease of separation. Tunable solvents are homogeneous mixtures of water or polyethylene glycol with organics such as acetonitrile, dioxane, and THF that can be used for homogeneously catalyzed reactions. Modest pressures of a soluble gas, generally CO_2_, achieve facile post-reaction heterogeneous separation of products from the catalyst. Examples shown here are rhodium-catalyzed hydroformylation of 1-octene and *p*-methylstyrene and palladium catalyzed C-O coupling to produce *o*-tolyl-3,5-xylyl ether and 3,5-di-*tert-*butylphenol. Both were successfully carried out in homogeneous tunable solvents followed by separation efficiencies of up to 99% with CO_2_ pressures of 3 MPa. Further examples in tunable solvents are enzyme catalyzed reactions such as kinetic resolution of *rac*-1-phenylethyl acetate and hydrolysis of 2-phenylethyl acetate (2PEA) to 2-phenylethanol (2PE). Another tunable solvent is nearcritical water (NCW), whose unique properties offer advantages for developing sustainable alternatives to traditional processes. Some examples discussed are Friedel-Crafts alkylation and acylation, hydrolysis of benzoate esters, and water-catalyzed deprotection of *N*-Boc-protected amine compounds.

## 1. Introduction

Catalysis plays a vital role in the chemical industry by contributing to both its economical success and environmental sustainability. More than 75% of all industrial chemical transformations employ catalysts in areas as diverse as polymers, pharmaceuticals, agrochemicals, and petrochemicals. In fact, 90% of newly developed processes involve the use of catalysts [1]. In addition, the growing focus on environmental conservation relies heavily on developments in the field of catalysis. Heterogeneous catalysis is widely used in industrial applications because of the facile separation, which often results in lower operating costs. On the other hand, homogeneous catalysis has limited industrial applications due to the difficult and costly catalyst separation and recovery. Table 1 compares homogeneous and heterogeneous catalysis in terms of catalytic effectiveness, catalyst properties, and catalyst separation [1]. Homogeneous catalysts offers improved selectivity, increased activity, and avoid mass transfer limitations, which may permit lower temperatures.

**Table 1:** Comparison between Homogeneous and Heterogeneous Catalysis.

	Homogeneous	Heterogeneous
**Active Centers**	All atoms	Only surface atoms
**Selectivity**	High	Low
**Mass Transfer Limitations**	Very rare	Can be severe
**Structure/Mechanism**	Defined	Undefined
**Catalyst Separation**	Tedious/Expensive (extraction or distillation)	Easy
**Applicability**	Limited	Wide
**Cost of Catalyst Losses**	High	Low

We review tunable solvents which combine homogeneous reactions and heterogeneous separations –both those activated by CO_2_ and nearcritical water (NCW). They are powerful tools for improving the operating conditions—e.g., lower temperatures and shorter reactions—of many chemical reactions, while reducing the amount of energy required and waste generated. Using these tunable solvents allows for homogeneous reactions followed by facile post-reaction heterogeneous separations. The separation of the products from the catalysts and solvents is achieved by manipulating the phase behavior with a trigger such as pressure or temperature. In the first part, the benefits and limitations of CO_2_-tunable solvents for hydroformylation and C-O coupling reactions as well as enzymatic catalyzed hydrolysis and kinetic resolution reactions will be discussed. The second part will focus on NCW-mediated reactions including Friedel-Crafts alkylation and acylation, *t*-Boc deprotection, and base-catalyzed hydrolysis. Note also that another powerful class of tunable solvents is supercritical fluids, also often used advantageously for reactions; however, these are reviewed elsewhere [2,3,4,5,6,7,8,9,10,11,12,13].

## 2. Tunable Solvents

**Figure 1:**
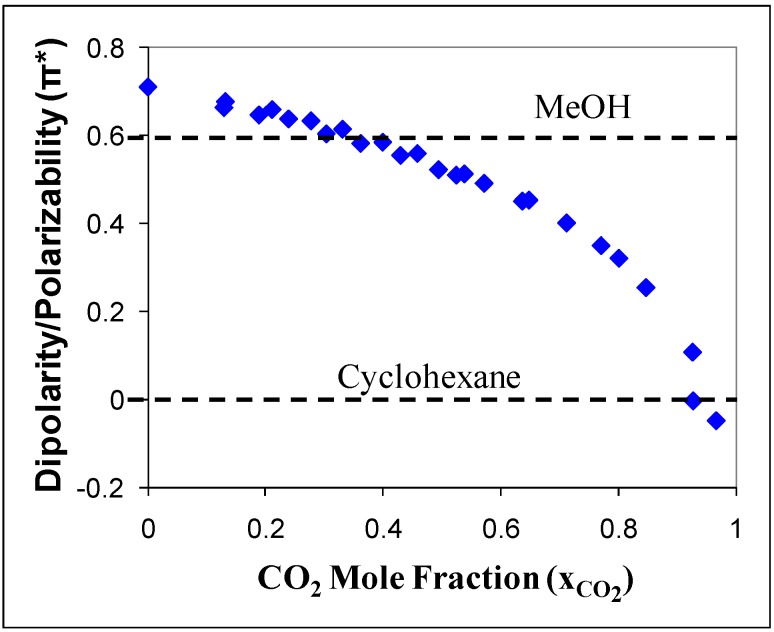
Kamlet-Taft dipolarity and polarizability (π*) of acetonitrile/CO_2_ mixture as a function of CO_2_ mole fraction [14].

Gas-expanded liquids (GXLs) result from the pressurized dissolution of a gas, such as CO_2_, into organics like THF or acetonitrile. The GXL's physical properties can be tuned with the composition of the mixture, *i.e.* the amount of antisolvent gas added to the organic. Ford *et al.* [14] reported the Kamlet-Taft solvatochromic parameters for CO_2_-expanded acetonitrile. The polarity/polarizability (π*) of the CO_2_-expanded acetonitrile was measured as a function of added CO_2_ in the mixture, as shown in Figure 1. Without CO_2_, acetonitrile has a greater polarity (π* = 0.75) than methanol (π* = 0.6). The dissolution of CO_2_ causes the polarity of the mixture to decrease gradually up to a CO_2_ mole fraction of 0.8 where a sharp decrease in π* towards a value of zero (π* = 0 for cyclohexane, π*= -0.1 for CO_2_). The polarity of the CO_2_-expanded liquid can be easily tuned by controlling the amount of gas added to the mixture.

**Figure 2:**
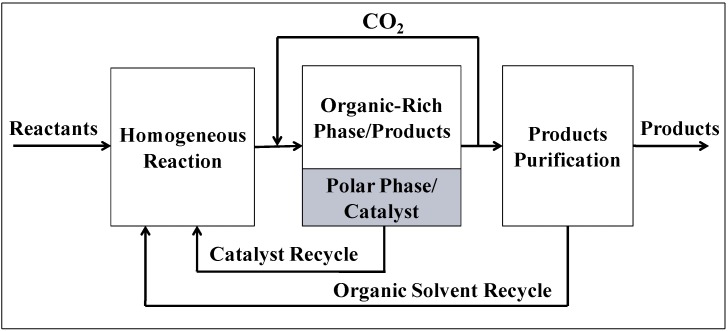
Schematic of an OATS Process.

Organic-Aqueous Tunable Solvents (OATS) consist of miscible mixtures of an aprotic organic solvent (some examples are 1,4-dioxane, acetonitrile, or tetrahydrofuran) and a polar protic solvent (water) [15]; tunable solvent mixtures are used as homogeneous reaction media. The mixture is chosen such that upon the addition of an antisolvent gas, e.g., CO_2,_ a phase split occurs yielding a biphasic liquid-liquid system. This takes place because CO_2_ is completely soluble in most organics but only slightly soluble in aqueous media. A schematic of the process using OATS is shown in Figure 2. This concept allows for sustainable design of chemical processes which recycle the catalyst and the process solvents; admittedly small make-up streams may be needed to compensate for solvent losses and catalyst leaching. The power of CO_2_-tunable solvents comes from the ability to benefit from increased reaction rates, improved yields and selectivity, and consistent catalytic activity while maintaining facile separation and recycle of the catalyst. OATS systems are a useful new tool for reaction engineering and catalysis.

The applications of OATS systems depend on their suitability for homogeneous reactions and on the efficient heterogeneous separations of the products from the catalyst. The applicability depends strongly on the phase equilibria. A successful solvent system provides a readily attainable phase split with a relatively large liquid-liquid region and asymmetric composition distribution to allow for facile separation of the products and recycle of the catalyst [16]. Thus the measurement and modeling of the thermodynamics of phase behavior is an essential part of this application.

Lazzaroni *et al.* [17] reported the high pressure phase behavior of various OATS mixtures. An example of the ternary phase behavior of the liquid-liquid system under CO_2_ pressure is shown in Table 2. Prior to the addition of CO_2_, a homogeneous mixture acetonitrile (ACN) and water (H_2_O) is introduced into a pressure cell and then CO_2_ – in the form of a pressurized gas – is added to induce a phase split. After equilibrium, the composition of the two liquid phases is measured. The mole fraction of CO_2_ does not exceed 4% in the aqueous-rich phase at pressures up to 5.2 MPa. As CO_2_ pressure is increased, the separation improves; less water is present in the organic-rich phase, and there is less organic is in the aqueous phase.

**Table 2:** Liquid-Liquid Phase Behavior of Acetonitrile-Water Tunable Solvents with CO_2_ [17].

**P (MPa)**	**Aqueous-Rich Phase**			**Acetonitrile-Rich Phase**		
	xCO_2_	xACN	xH_2_O	xCO_2_	xACN	xH_2_O
1.9	0.04	0.23	0.73	0.08	0.44	0.49
2.4	0.02	0.14	0.85	0.17	0.59	0.24
3.1	0.01	0.07	0.92	0.26	0.62	0.12
4.1	0.01	0.08	0.91	0.41	0.53	0.07
5.2	0.03	0.06	0.92	0.50	0.43	0.07

Hydroformylation of hydrophobic aromatic and aliphatic compounds was used as a model reaction in OATS. Hydroformylation is particularly relevant as a proof of concept since it is conventionally carried out in biphasic aqueous-nonpolar organic systems [1]. The reaction takes place in the aqueous phase in the presence of the water-soluble rhodium-triphenylphosphine catalytic complex. Biphasic hydroformylation works well for up to C_4_ alkenes as their water solubility is sufficient [18,19] (for example the solubility of propene is 200 ppm [20,21]. However; hydroformylation of longer chain and aromatic alkenes is not possible due to their exponentially decreasing solubility in water (the solubility of 1-octene is only 2.7 ppm [20,21]). Although the low aqueous solubility of higher alkenes is perceived as a disadvantage for a biphasic process, it is an opportunity for OATS-mediated process.

**Scheme 1:**
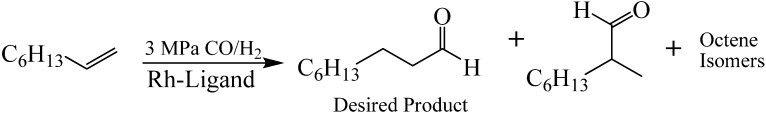
Hydroformylation of 1-octene to 1-nonanal and side products.

Hallett *et al.* [22] reported the Rh catalyzed hydroformylation of 1-octene in tetrahydrofuran (THF)-H_2_O OATS with two hydrophilic ligands: monosulfonated triphenylphosphine (TPPMS) and trisulfonated triphenylphosphine (TPPTS). The reaction was carried out at 3 MPa of syngas pressure (1:1 moles of H_2_:CO) as shown in Scheme 1. 

**Figure 3:**
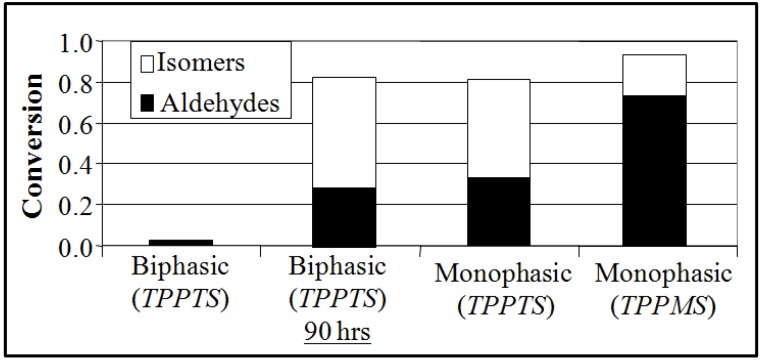
Conversion and yield of 1-octene hydroformylation at 120 °C and 1 h (except where noted) in monophasic THF-H_2_O and biphasic (no THF) solvents with Rh:ligand:substrate ratio of 1:10:500 [22].

The homogeneous reaction rate in OATS was approximately two orders of magnitude greater than the biphasic reactions as shown in Figure 3, the linear-to-branched product ratio and the turnover frequencies (TOF) are 2.8 and 115 for TPPTS and 2.3 and 350 for TPPMS, respectively. TPPTS showed less yield of aldehydes than TPPMS, which was attributed to the electronic effects of the sulfonate groups. 

**Figure 4:**
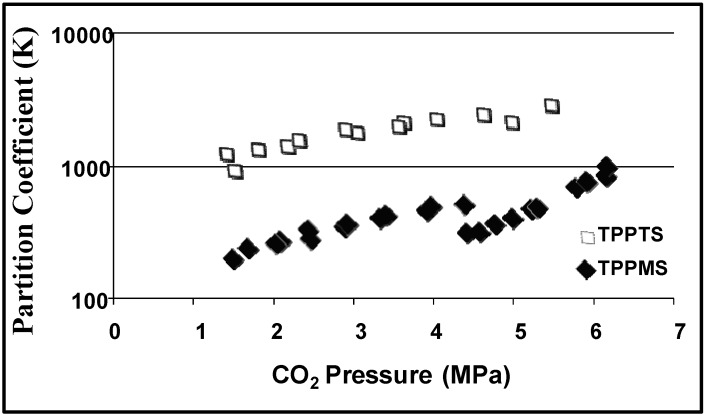
Partitioning of hydrophilic TPPMS and TPPTS in the aqueous phase as a function of CO_2_ pressure in THF/H_2_O (70:30 v:v) at 25 °C [23].

**Figure 5:**
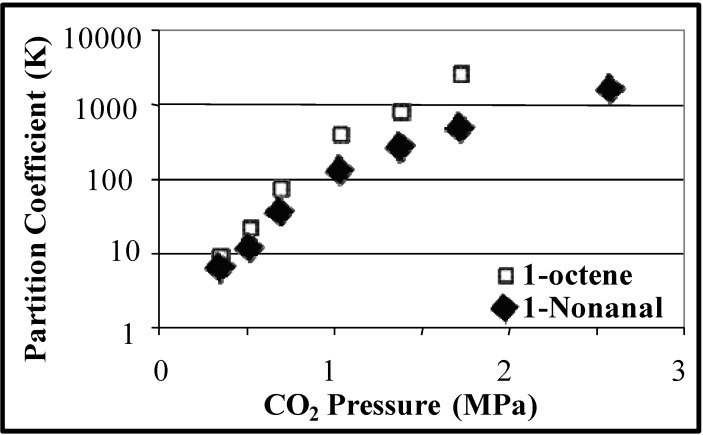
Partitioning of hydrophobic 1-octene and nonanal in the CO_2_-expanded organic phase as a function of CO_2_ pressure in THF/H_2_O (70:30 v:v) at 25 °C [23].

Partition coefficients are used to evaluate the efficiency of the CO_2_-induced heterogeneous separations. The partition coefficient (K) is defined as the ratio of the concentration of the substance (reactants, products, or catalyst) in the desired phase (e.g., aqueous phase for the hydrophilic catalyst) to the concentration of the substance in the undesired phase (e.g., the organic rich phase for the hydrophilic catalyst). Figure 4 shows the partition coefficients of 1-octene and 1-nonanal as a function of CO_2_ pressure and Figure 5 shows the partition coefficient of the ligands TPPTS and TPPMS as a function of CO_2_ pressure [23]. The partitioning of the ligands in the aqueous phase and the partitioning of the product in the organic phase increases as CO_2_ pressure increases due to the improved phase separation at higher pressures. TPPTS partitions better into the aqueous phase when compared to TPPMS due to the larger number of sulfonate groups, which improves its hydrophilic nature. Nonetheless, more than 99.9% of both ligands partitions in the aqueous phase with the moderate CO_2_ pressure of 3 MPa. It should be noted that the reaction itself is carried out under 3 MPa of CO/H_2_ pressure, and therefore; the 3 MPa of CO_2_ pressure required for the separation is easily implemented. The recycle of the catalyst for three consecutive reactions was demonstrated with consistent catalytic activity and turnover frequencies (TOF) of 51 ± 3 h^-1^ and rhodium leaching of less than 1 ppm in the organic phase, as determined by atomic absorption spectroscopy. 

**Scheme 2:**
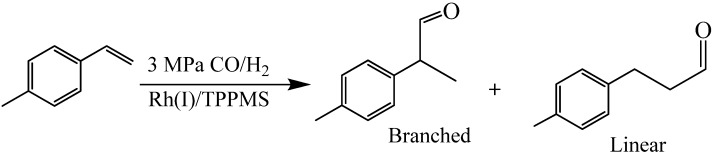
Hydroformylation of *p*-methylstyrene in OATS.

**Figure 6:**
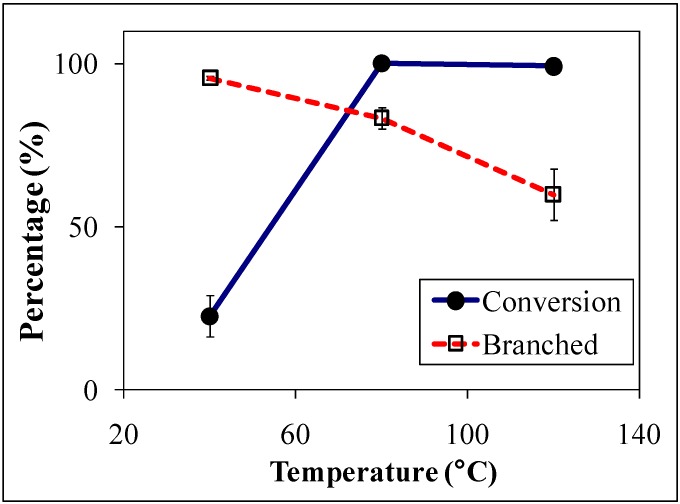
Conversion and branched product yield of *p*-methylstyrene hydroformylation in ACN/H_2_O with 30 bar syngas pressure and catalyst:ligand:substrate ratio of 1:7:400 [15].

Blasucci *et al.* [15] reported the hydroformylation of *p*-methylstyrene (Scheme 2) in OATS systems containing acetonitrile and water. The reaction produces linear and branched aldehydes with the branched aldehyde (2-*p*-tolylpropanal) as the desired product. 2-*p*-tolylpropanal is a mimic molecule for 2-*p*-isobutylbenzenepropanal, which is an intermediate in the synthesis of ibuprofen [24,25] — a nonsteroidal anti-inflammatory drug with annual demand of more than 12 million kg per annum [26]. The starting material conversion and branched product yield are shown in Figure 6. The TOF increases from 92 at 40 °C to 406 at 80 °C and the yield of the branched product decreased from 95% at 40 °C to around 80% at 80 °C. 

**Figure 7:**
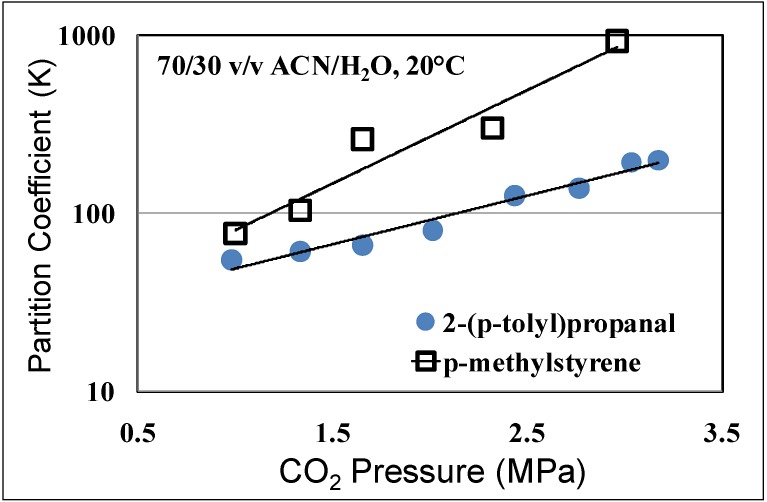
Partitioning of hydroformylation reaction substrates in the organic-rich phase as a function of CO_2_ pressure in ACN/H_2_O (70:30 v:v) at room temperature [15].

The reduction in the yield of the branched product is attributed to the β-hydride elimination [27]; the intermediate complex of the Rh with the branched product is converted back into the starting material at higher temperatures. The reaction rates and selectivities of styrene hydroformylation in OATS show at least an order of magnitude improvement over heterogeneously reported systems using solid supports [28] and reactions run with ionic liquid modified silica sol-gel [29]. The partitioning coefficient of the starting material and the branched product were also reported in acetonitrile/H_2_O OATS at pressures between 1 MPa and 3.5 MPa as shown in Figure 7. The partition coefficient of 2-*p*-tolylpropanal increased from 50 at 1 MPa of CO_2_ to 200 at 3 MPa of CO_2_; more than 99% of the desired product partitions in the acetonitrile-rich phase at moderate pressures of 2.5 MPa.

In addition, OATS systems have been used for enzyme-catalyzed reactions. Enzymes function at moderate temperatures and provide synthetic approaches that may otherwise require multiple steps using less selective and active metal-catalysts. However, their applications are largely limited to aqueous media, which constrains the possibility of using them for hydrophobic substrates [30]. OATS provide a suitable medium for enzymatic transformation of hydrophobic substrates and eliminate the need for enzyme immobilization since heterogeneous separation and recovery can be readily achieved.

Carrying out enzymatic reactions in CO_2_-OATS requires the use of a buffer to maintain an enzyme-friendly environment, and even then only some enzymes retain adequate activity in the mixed solvent. The pH of buffered and unbuffered water/dioxane mixtures as a function of CO_2_ pressures were reported [30]; the pH of unbuffered dioxane/H_2_O (30/70 v/v) was 3 with less than 1 MPa of CO_2_. However; the presence of a sodium phosphate monobasic monohydrate (phosphate) buffer maintains the solution pH above 6 for CO_2_ pressures of up to 4 MPa. Hill *et al.* [31] reported the effect of phosphate and (2-hydroxyethyl)-1-piperazineethanesulfonic acid (HEPES) buffers on the phase behavior of dioxane-H_2_O-CO_2_, and both increase the amount of water in the CO_2_-expanded dioxane. For example, the dioxane-rich phase contains about 10% water at 3.7 MPa; in the presence of 150 mM phosphate the amount of water in the GXL increases to 15% at 3.8 MPa and in the presence of 150 mM HEPES the amount of water increases to 50% in the GXL at 3.8 MPa. The need for buffer can be avoided by using gases other than CO_2_ as a homogeneous to heterogeneous trigger. Blassuci *et al.* [15] reported the use of propane in THF/H_2_O tunable solvent systems at 303 K and 313 K and pressures range of 0.43 to 1.35 MPa, as shown in Table 3. 

**Table 3:** Liquid-liquid equilibria of H_2_O(1)/propane(2)/ THF(3) OATS at 30 °C [15].

*P*(MPa)	H_2_O-rich phase			THF-rich phase		
	x_1_	x_2_	x_3_	x_1_	x_2_	x_3_
0.43	0.706	0.008	0.286	0.061	0.083	0.856
0.56	0.711	0.006	0.283	0.040	0.175	0.785
0.80	0.776	0.016	0.208	0.031	0.310	0.659
0.92	0.820	0.024	0.156	0.028	0.452	0.520

**Scheme 3:**
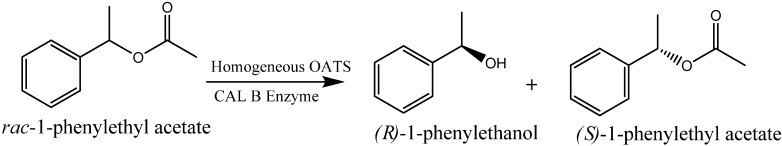
Kinetic Resolution of *rac*-1-phenylethyl acetate to *(R)-*1-phenylethanol using CAL B.

**Scheme 4:**
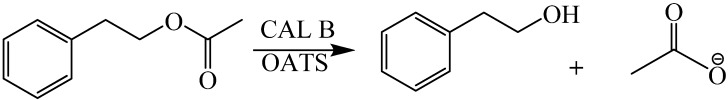
Hydrolysis of 2-phenylethyl acetate to 2-phenylethanol.

These systems offer two notable benefits over CO_2_-induced OATS: an improved phase separation at lower pressures and the elimination of in situ carbonic acid formation. For example, the amount of water in the propane-expanded THF is 3 wt% at 0.8MPa compared to 9 wt% H_2_O in the CO_2_-expanded THF at 4MPa. The use of flammable propane comes with its procedural limitations and thus; a balance of benefits and limitations is needed. Hill *et al.* [31] reported the kinetic resolution of *rac*-1-phenylethyl acetate to *(R)-*1-phenylethanol using *Candida Antarctica* lipase B (CAL B) (Scheme 3) in OATS systems. The substrate saturation and pseudo 1st order rate constants are shown in Table 4. The highest substrate saturation and rate constant were observed in phosphate-buffered 1,4-dioxane-H_2_O (30:70 by volume) OATS systems. This is a powerful example of the tunability of OATS mixtures; one must optimize both the catalytic activity and separation efficiency to maximize the benefits of tunable solvents. In addition, Broering *et al.* [30] reported the use of sodium phosphate monobasic monohydrate (phosphate) buffered aqueous/1,4-dioxane tunable solvents for the CAL B catalyzed hydrolysis of 2-phenylethyl acetate (2PEA) to 2-phenylethanol (2PE), as shown in Scheme 4. 

**Table 4:** Kinetic Parameters for CAL B Kinetic Resolution of *rac*-1-phenylethyl acetate to *(R)-*1-phenylethanol at room temperature [31].

Solvent	Volume% in OATS	Substrate Saturation (mM)	Pseudo 1st Order Rate Constant (1/sec)	Product Enantiomeric Excess (ee)
Acetone	30	18.1 ± 0.9	0.003 ± 0.001	> 99%
Acetonitrile	30	9.1 ± 0.5	0.009 ± 0.001	> 99%
(1,4)-Dioxane	30	17.7 ± 0.8	0.014 ± 0.001	> 99%

**Figure 8:**
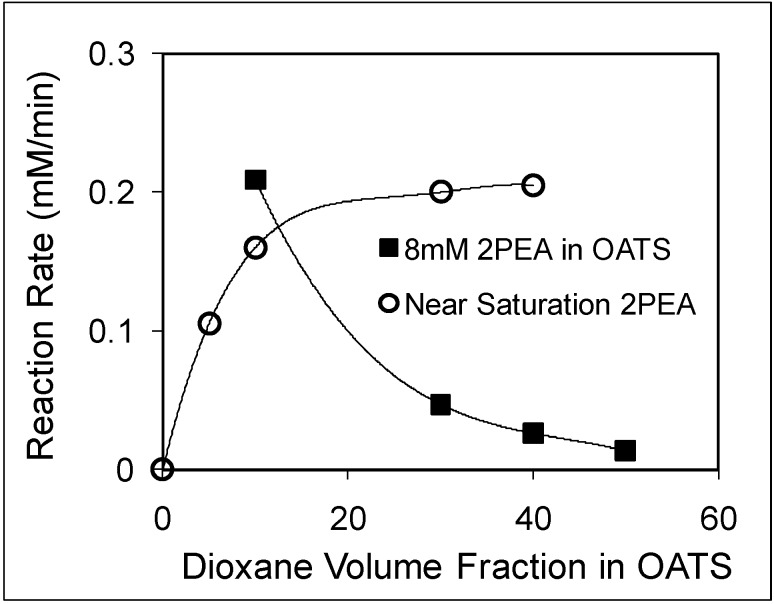
Monophasic Ester Hydrolysis Reaction Rates for 8mM (+) and Near-Saturation (O) of 2PEA Solutions as a Function of 1,4-dioxane Fraction in dioxane/H_2_O at room temperature [30].

The reaction rates with 8 mM and near-saturation of 2PEA concentrations as a function of increased dioxane volume fraction are shown in Figure 8. The limited solubility of 2PEA in water results in a slow reaction with negligible conversion to products. The addition of dioxane reduces the reaction rate of the 8 mM PEA solution from 0.2 mM/min at 10 vol% dioxane to 0.05 mM/min at 30 vol% dioxane. 

**Figure 9:**
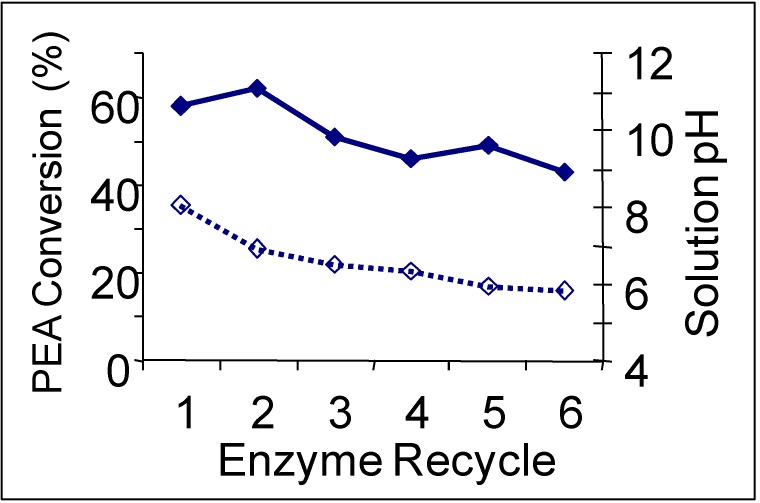
CAL B Recycles for Ester Hydrolysis in dioxane/H_2_O (40:60 v:v) (conversion 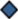 and measured solution pH 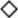) [30].

The reduction in enzyme activity could have resulted from enzyme poisoning and/or deactivation. However, the solubility of 2PEA improves drastically in the presence of dioxane, which compensates for the deactivation of the enzyme and causes the near-saturated 2PEA reaction rate to improve from 0.15 mM/min at 10% dioxane to 0.22 mM/min at 40% dioxane. The 40% dioxane mixture was chosen to recycle the enzyme because it provided the highest specific rate for the reaction and because the volume of the organic layer is larger, which is favorable for product removal. The enzyme was recycled for six consecutive reactions with an average conversion of 61% over two hours. CO_2_ was applied after each reaction to induce heterogeneous separation of the product; 80% of the 2PE was removed by decanting. The enzymes maintained a catalytic activity of about 85% after six cycles, as shown in Figure 9. The decrease in enzymatic activity is explained by dilution of the mixture due to sampling, which accounts for 11% activity loss, as well as enzyme deactivation due to reduction in solution pH resulting from in situ carbonic acid formation during CO_2_ induced phase separation and accumulation of acetate in the aqueous phase despite the use of 150 mM phosphate buffer.

PEG tunable solvent systems (POTS) allow carrying out reactions that may not be possible in OATS. PEG is considered a green solvent due its low toxicity, negligible vapor pressure, and biodegradability [32]. In some cases POTS offers advantages over OATS, as PEG is miscible with most organics and can complex with cations and activates anions in reactions containing salts, acting both as medium and contributor for the reaction [33]. POTS can advantageously combine homogeneous reactions with heterogeneous separations. Like OATS systems, the hydrophilic catalyst is retained in the PEG-rich phase and the products partitioned preferentially in the GXL from which they can be easily isolated. Donaldson *et al.* [34] reported the ternary phase behavior of polyethylene glycol 400 (PEG) and CO_2_ with 1,4-dioxane and acetonitrile. However, unlike water, PEG dissolves a substantial amount of CO_2_. As an example, the PEG-rich phase contains 49.9 wt% PEG, 16.4 wt% dioxane, and 33.7 wt% CO_2_ at 5.24 MPa and 298K. At the same conditions, the dioxane-rich phase contains 74.1 wt% CO_2_, 22.5 wt% dioxane, and only 3.4% PEG. The amount of PEG in the dioxane-rich phase makes this system suitable for catalyst separation and recycles. 

**Scheme 5:**
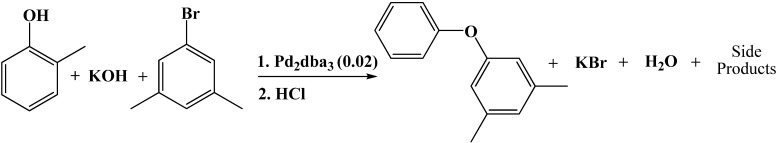
Reaction of 1-bromo-3,5-dimethylbenzene and *o*-cresol with potassium hydroxide to produce *o*-tolyl-3,5-xylyl ether.

**Scheme 6:**
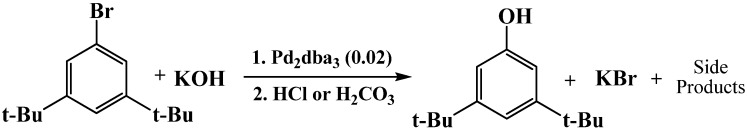
Reaction of 1-bromo-3,5-di-*tert*-butylbenzene with potassium hydroxide to produce 3,5-di-*tert*-butylphenol.

Blasucci *et al.* [15] reported two reactions in POTS—the C-O coupling reaction of 1-bromo-3,5-dimethylbenzene and *o*-cresol with potassium hydroxide to produce *o*-tolyl-3,5-xylyl ether (Scheme 5) and the reaction of 1-bromo-3,5-di-*tert-*butylbenzene and potassium hydroxide yielding 3,5-di-*tert-*butylphenol (Scheme 6) in PEG, PEG/dioxane, PEG/dioxane/water solvent mixtures (Table 6 and Table 7). The PEG/dioxane/H_2_O solvent system showed improved conversion and desired product selectivity for both the ether (Scheme 5) and the phenol (Scheme 6). CO_2_ pressure induces heterogeneous separation of the products. 

**Table 6:** Conversion of 1-bromo-3,5-dimethylbenzene and *o*-cresol with potassium hydroxide and selectivity of *o*-tolyl-3,5-xylyl ether in different solvent systems at 80°C [15].

Solvent System	Conversion (%)	Selectivity(%)
PEG 400	60 ± 6	65 ± 2
PEG 400 (72 wt)% / 1,4-Dioxane (28 wt%)	63 ± 9	64 ± 5
PEG 400 (60 wt%) / 1,4-Dioxane (24 wt%) / Water	80 ± 10	71 ± 2

**Table 7:** Conversion of 1-bromo-3,5-di-*tert*-butylbenzene with potassium hydroxide and selectivity of 3,5-di-*tert*-butylphenol in different solvent systems 80 °C [15].

Solvent System	Conversion (%)	Selectivity (%)
PEG 400	100 ± 0	60 ± 6
PEG 400 (72 wt)% / 1,4-Dioxane (28 wt%)	100 ± 0	44 ± 4
PEG 400 (60 wt%) / 1,4-Dioxane (24 wt%) / Water	80 ± 7	68 ± 2

**Figure 10:**
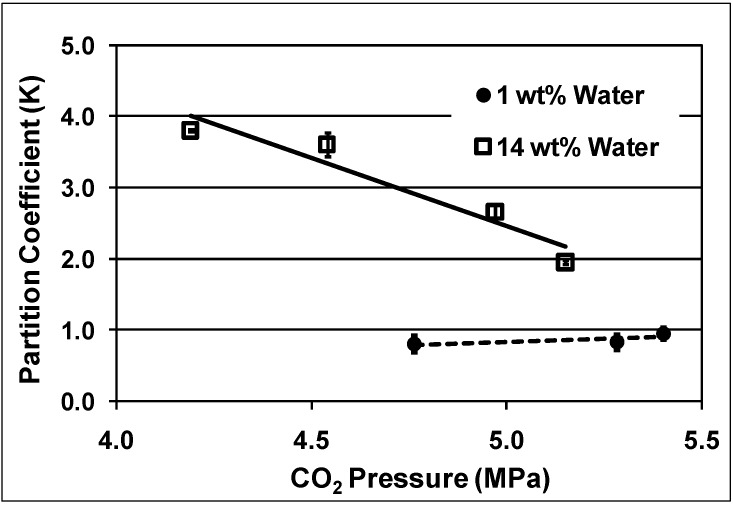
Partitioning of *o*-tolyl-3,5-xylyl ether in the PEG-rich phase as a function of CO_2_ pressure and water contents at room temperature [15].

The partitioning of *o*-tolyl-3,5-xylyl ether between the dioxane-rich phase and the PEG-rich phase as a function of CO_2_ pressure and water content is shown in Figure 10. The presence of water resulted in decreasing the amount of product in the PEG phase, increasing the partition coefficient from 1 with 1 wt% water to more than 3 with 14 wt% water at 4.7 MPa. Two effects that might explain such a result are the increased polarity of the PEG-rich phase or hydrogen bonding between PEG and water, which could disrupt the interaction between PEG and the solute molecule. Similar trends were obtained for 3,5-di-*tert-*butylphenol and the Pd catalyst. Increased CO_2_ pressure improved retaining the Pd catalyst in the PEG phase but decreased the products recovery into the organic phase. 

## 3. Nearcritical Water (NCW)

NCW is liquid water at elevated temperatures in the range of 200-350 °C and at saturation pressure, yet still below the critical point at 374 °C and 22.1 MPa. NCW exhibits many solvent properties similar to polar organic solvents such as acetone [35,36,37,38,39]. For example, the density of water decreases from 1.0 g/cc at room temperature to 0.7 g/cc at 275 °C [40]. Similarly, the dielectric constant decreases from 78 at ambient conditions to 20 at 275 °C [41]. The decrease in dielectric constant is attributed to diminishing hydrogen bonding in NCW [42,43]. As a result, a significant improvement in the solubility of nonpolar organics such as toluene is achieved at nearcritical conditions. In addition, the increased dissociation constant of water at nearcritical conditions offers exciting opportunities for reversible in-situ acid and base catalysis. The dissociation constant—which is the ability of water to ionize into H^+^ and OH-—increases three orders of magnitude from 10^-14^ at 25 °C to about 10^-11^ at 275 °C [44]. Therefore, NCW can act as a reversible in-situ acid or base catalyst. This offers many advantages to develop sustainable processes since NCW acts as a reversible acid or base catalyst, avoiding the need for acids and bases, thus eliminating waste-intensive neutralization steps.

The opportunities for carrying out homogeneous catalysis in NCW stem from the increased solubility of organic substrates—especially nonpolar—at elevated temperatures, coupled with the still very good solubility of salts. The enhanced solubility of organics facilitates the use of homogeneous catalysis, allowing improved reaction rates and enhanced yields. The especially simple post-reaction separation of the products—mere cooling—reduces cost and waste-intensive separation strategies. The organic phase is simply decanted and more starting materials are added for the consecutive reaction cycle. The use of water as a solvent and the ability to recycle the catalyst make NCW a sustainable medium for combining homogeneous reactions with heterogeneous separations.

**Figure 11:**
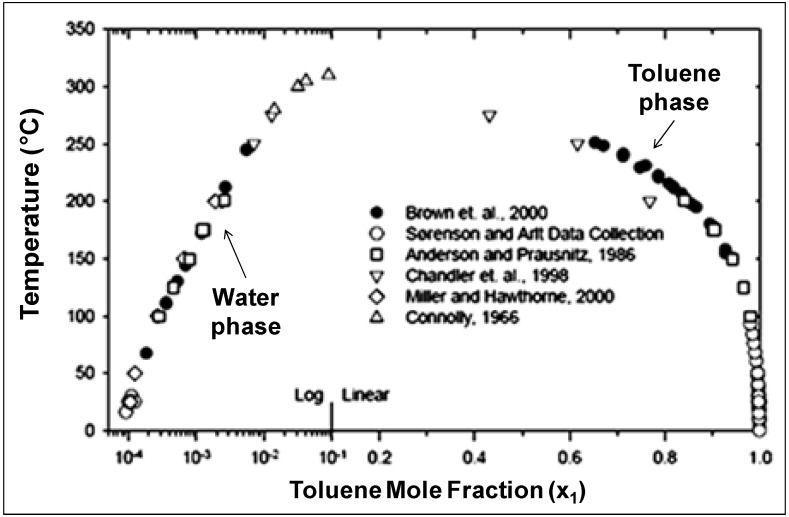
Liquid-liquid equilibria of toluene (component 1) and water (component 2). The data above 100 °C were reported at the saturation pressure [45,46,47,48,51,58].

**Scheme 7:**
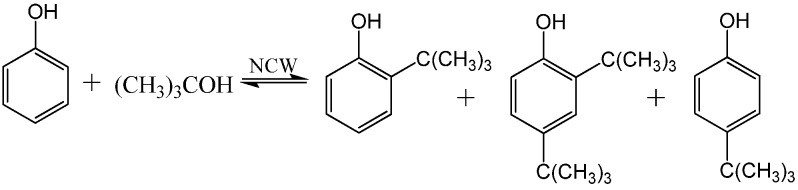
Alkylation of phenol with *tert*-butanol to produce 2-*tert*-butylphenol, 2,4-di-*tert*-butylphenol, and 4-*tert*-butylphenol.

The solubility of many organics in NCW has been reported [45,46,47,48,49,50,51,52,53,54,55,56,57]. The effect of temperature on solubility is tremendous; benzene’s solubility increases from 500 ppm at ambient conditions to complete miscibility at 305 °C [53] and *n*-hexane solubility increases by almost five orders of magnitude from ambient water to NCW [48]. The ease of separating organics from NCW upon cooling can be demonstrated by examining the solubility curve of toluene-water system, represented in Figure 11 [45,46,47,48,51,58]. Toluene has limited solubility in water at ambient conditions with a mole fraction of 0.0001 at 25 °C. As the mixture is heated, the solubility increases exponentially up to a mole fraction of 0.014 at 280 °C and even more rapidly up to the UCST at 310 °C. On the other hand, the solubility of water in the toluene phase is 0.0025 at ambient temperature and it increases linearly as the mixture is heated. The log-linear solubility relationship is common for nonpolar/H_2_O pairs [40]. The finite solubility and favorable phase separation at room temperature provides excellent opportunities for product recovery, catalyst recycle, and easy processing of chemicals. NCW has been used for many reactions and comprehensive reviews are available [35,38,39,40,59,60,61,62,63,64]. We choose to discuss selective and representative examples, which take advantage of NCW's unique properties for coupling reaction and separation. Chandler *et al.* [65] reported the use of NCW as an acid catalyst for Friedel-Crafts alkylation reactions, which eliminates the need for the usual aluminum chloride (AlCl_3_) acid catalyst, which is corrosive, difficult to handle, and often used greater than stoichiometric amounts. The increased hydronium ion concentration at 275 °C catalyzed the reaction of phenol with *tert*-butyl alcohol to the products 2-*tert*-butylphenol, 4-*tert*-butylphenol, and 2,4-di-*tert*-butylphenol as shown in Scheme 7. 

**Figure 12:**
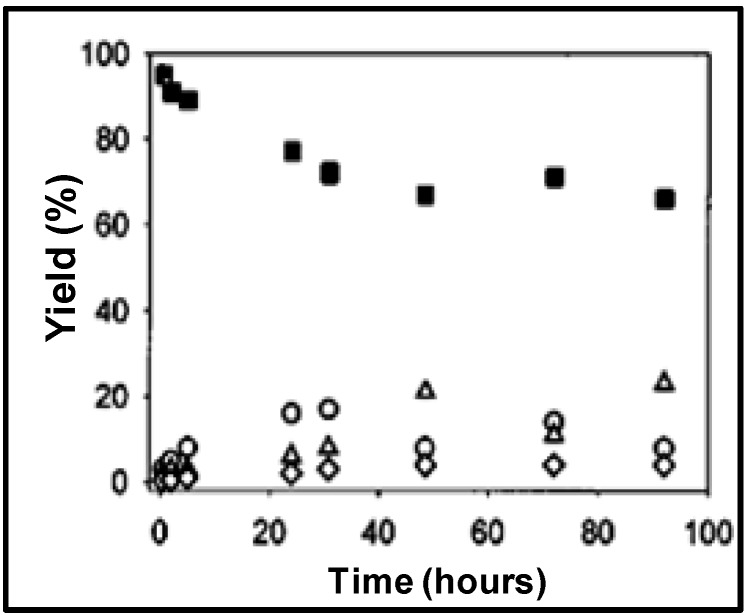
Molar yield of phenol (■) alkylation with *tert*-butanol to the products 2-*tert*-butylphenol (○), 4-*tert*-butylphenol (Δ), and 2,4-di-*tert*-butylphenol (◊) at 275 °C [65].

The concentration of the 2-*tert*-butylphenol reached 17% molar yield after 30 hours of reaction time and then decreases to the equilibrium concentration of 10%. On the other hand, 4-*tert*-butylphenol increased linearly to 20% after 50 hours of reaction time indicating thermodynamic control of the product formation. The equilibrium products distribution, shown in Figure 12, is similar to acid-catalyzed reactions reported [66]. However and in contrast with the NCW-mediated reaction, five times excess of HClO_4_ is required in the conventional processes. The reaction of *p*-cresol with *tert*-butyl alcohol proceeds faster than that of phenol in NCW. It reaches equilibrium shortly after 1 hour of reaction time at 275 °C with 2-*tert*-butyl-4-methylohenol as the only product with 20% equilibrium concentration. The alkylation of phenol with isopropanol was also reported; the reaction yielded 2-isopropylphenol and 2,6-di-isopropylphenol with molar yields of about 20% and 10%, respectively, after 120 hours. Chandler *et al.* [67] reported the rate constants of phenol and *p*-cresol alkylation with *tert-*butanol at 250, 275, and 300 °C. The rate constants of both reactions increase by approximately an order of magnitude as the temperature is increased by 50 °C. 

**Figure 13:**
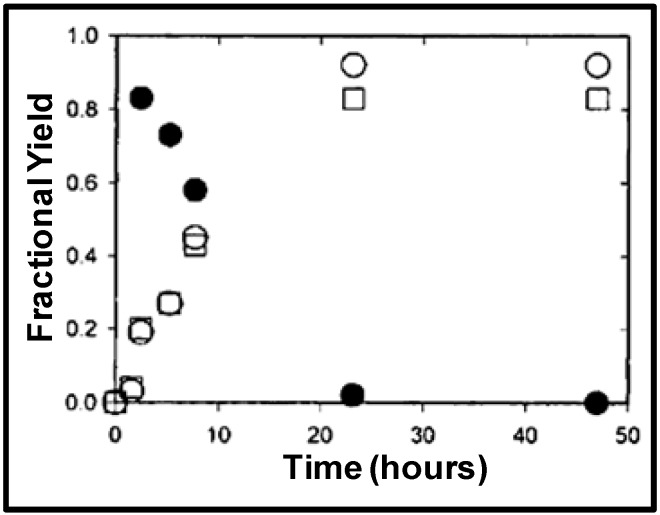
Decomposition of 2,4-dihydroxyacetophenone (●) to resorcinol (□) and acetic acid (○) in water at 250 °C [68].

**Figure 14:**
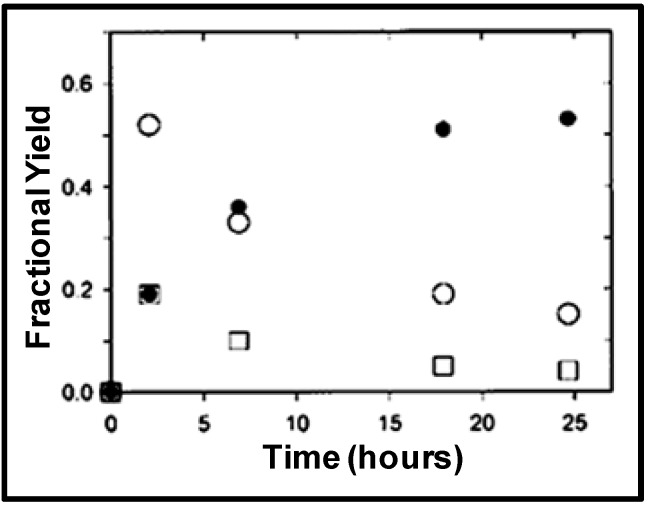
Acylation of resorcinol with acetic acid to products 2,4-dihydroxyacetophenone (●) resorcinol monoacetate (○), and resorcinol diacetate (□) in acetic acid at 290 °C [68].

NCW-mediated Friedel-Crafts acylation potentially eliminates the use of mineral or Lewis acids and as a result the subsequent neutralization steps that generate 5-10 lb of waste salt for each pound of product are unnecessary [36]. Brown *et al.* [68] reported the acid-free acylation of phenol and resorcinol using acetic acid in nearcritical water as well as in nearcritical acetic acid in the temperature range of 250-300 ºC. Phenol was primarily converted to 2-hydroxyacetophenone, 4-hydroxy-acetophenone, and phenyl acetate in approximately equal amounts, with a combined equilibrium yield of less than 1%, in aqueous acetic acid at 290 °C. Under the same conditions, resorcinol was converted to primarily 2,4-dihydroxyacetophenone with an equilibrium yield of 4%. The unfavorable equilibrium yields of desired acetylated products are due to their reversal at high temperatures in aqueous conditions. Figure 13 shows the reversal reaction of 2,4-dihydroxyacetophenone to resorcinol and acetic acid in water at 250 °C; near-complete decomposition of the 2,4-dihydroxyacetophenone is observed at 250 °C. To evaluate the effect of water on the reaction equilibrium, the acylation of phenol and resorcinol were run in acetic acid at 290 °C. Phenol was converted to 2-hydroxyacetophenone, 4-hydroxyacetophenone, and phenyl acetate with a combined equilibrium yield of 8 mol% and with 2-methylchromone and 4-methylcoumarine as by products. Under the same conditions, resorcinol was converted to primarily 2,4-dihydroxyacetophenone, with an equilibrium yield of more than 50 mol% in about 15 hours, details shown in Figure 14. The acid stabilizes the acylation products and improves the yield of the reaction at higher temperatures.

**Scheme 8:**
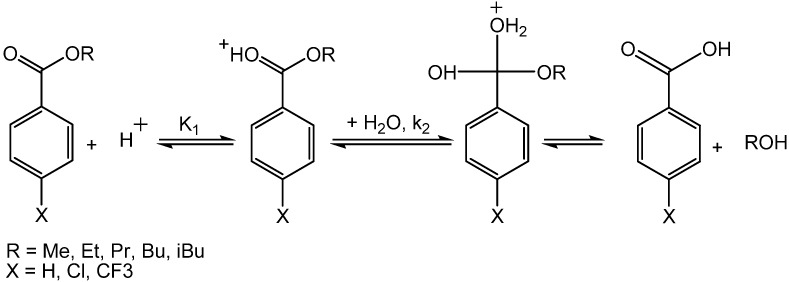
Acid catalyzed hydrolysis of benzoate esters in NCW.

**Figure 15:**
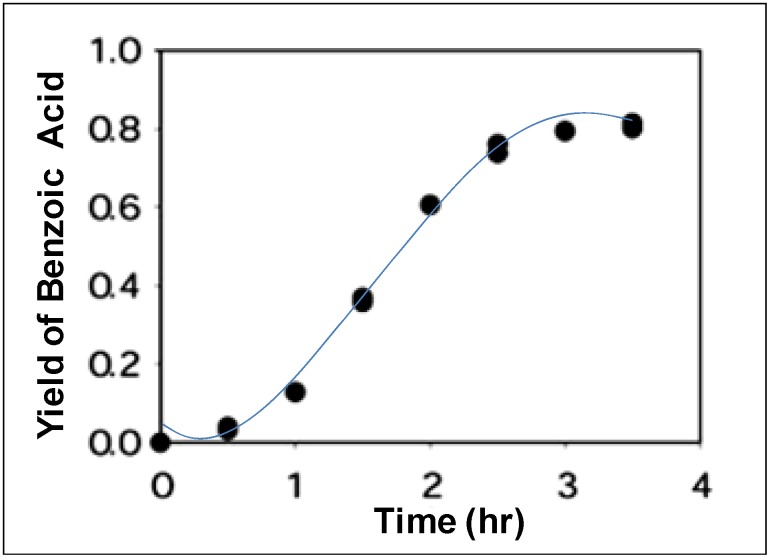
Conversion of *n*-propyl benzoate to benzoic acid and propanol in NCW at 250 °C [70].

NCW was used for the hydrolysis of various esters to produce the corresponding acids and alcohols in NCW [37,69,70,71]. For example, Lesutis *et al.* [70] showed the hydrolysis of benzoate esters in NCW (Scheme 8). The conversion of *n*-propyl benzoate to benzoic acid and propanol as a function of time at 250 °C is shown in Figure 15; the initial reaction is slow with 5% conversion after 30 minutes. The conversion increases exponentially after one hour to plateau at 80% conversion after three hours. The S-shaped curve suggests an autocatalytic mechanism; the autocatalytic acid-catalyzed hydrolysis of esters is modeled as shown below:

dx/dt = k[ester][H_2_O][H^+^]

where k = k_2_*K_1_, K_1_ is the equilibrium constant for the protonation of the ester, and k_2_ is the rate constant for the addition of water to the protonated ester. The water addition to the protonated ester is assumed to be the rate-controlling step. Table 8 shows the calculated rate constants with K_1_* as the equilibrium constant for the protonation of the unsubstituted ester. The concentration of protons is determined from the dissociation constants of water and the particular benzoic acid at 250 °C and the dissociation constants for substituted benzoic acids are calculated from the Hammett relationship using a ρ-value of 1.02. The rates of hydrolysis decrease as the length of the alcohol on the ester increases, due to steric hindrance. For example, k_2_K_1_* for methyl substituted benzoate ester is 26.9 L^2^/mol^2^ h and it decreases to 17.1 L^2^/mol^2^ h for *n*-butyl-substituted benzoate ester. Further decrease is observed for the iso-butyl substituted benzoate ester with k_2_K_1_* of 6.7 L^2^/mol^2^ h. The rate of hydrolysis of substituted isobutyl benzoates is independent of the substituent with ρ-value of about zero; the value of ρ is consistent with acid-catalyzed ester hydrolyses in aqueous solvents at lower temperatures. 

**Scheme 9:**
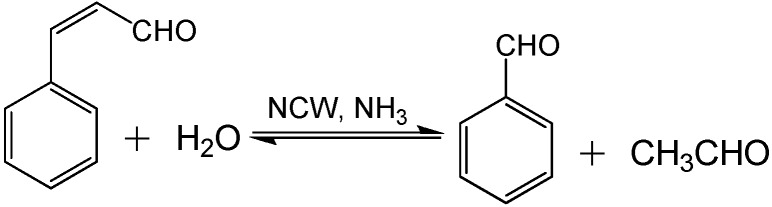
Cinnamaldehyde hydrolysis and benzaldehyde condensation.

**Figure 16:**
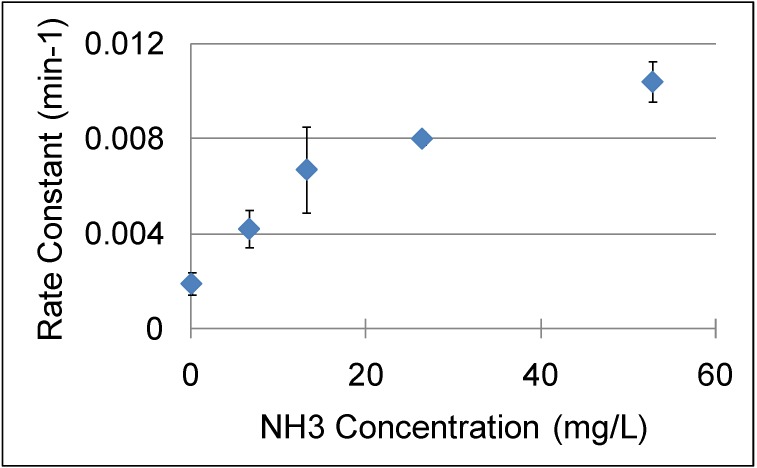
Evaluated rate constants of cinnamaldehyde hydrolysis to benzaldehyde with different NH_3_ concentrations and 240 °C [72].

NCW is also used for base catalysis; Lu *et al.* [72] reported the use of NCW and NH_3_-enriched NCW for hydrolysis of cinnamaldehyde and condensation of benzaldehyde (Scheme 9). Adding various amounts of NH_3_ ranging between 0-52.8 mg/L to NCW at 240 °C and 15MPa enhanced the rate constants of cinnamaldehyde hydrolysis by ten times as shown in Figure 16. Also, the yield of benzaldehyde improved by 30% upon the addition of NH_3_; the maximum yield of benzaldehyde obtained was 50% after three hours at 240 °C with 52.8 mg/L NH_3_. Side products such as benzyl alcohol, benzoic acid, styrene, and higher molecular weight compounds are also formed in small quantities. The increase in [OH^-^] upon the addition of NH_3_ improved the reaction rate under basic conditions. The reported data contains considerable deviations but an overall trend can be observed.

**Table 8:** Rate constants for the hydrolysis of *para*-substituted benzoate esters at 250 °C [70].

R-group	Substituent (X)	k_2_K_1_*(L^2^/mol^2^h)
Me	H	26.9 ± 2.5
Et	H	25.7 ± 0.9
Pr	H	10.4 ± 0.5
Bu	H	17.1 ± 0.6
Bui	H	6.7 ± 0.3
Bui	Cl	7.4 ± 0.6
Bui	CF3	7.0 ± 0.5

**Figure 17:**
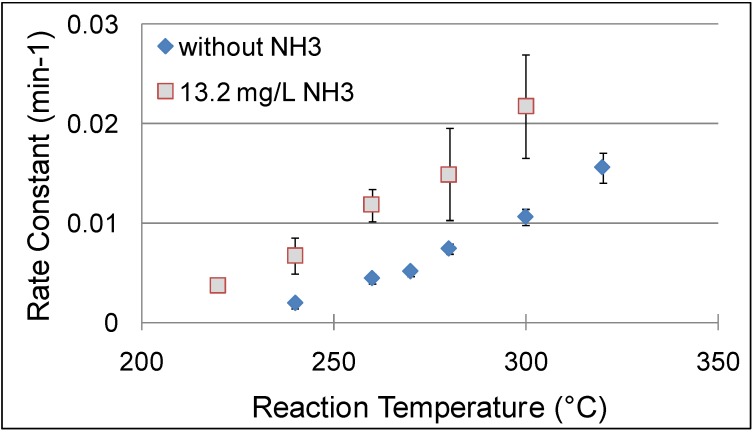
Evaluated rate constants of cinnamaldehyde hydrolysis to benzaldehyde at different temperatures and NH_3_ concentrations [72].

Also, data were reported for the effect of temperature on the hydrolysis of cinnamaldehyde at different temperatures between 220-320 °C with and without NH_3_; the addition of 13.2 mg/L of NH_3_ doubles the reaction rate in this temperature range as shown in Figure 17. In neat NCW, the conversion of cinnamaldehyde to benzaldehyde and acetaldehyde reaches 90% at 300 °C after three hours. With 13.2 mg/L of NH_3_, 90% conversion of the cinnamaldehyde is observed at 260 °C in less than three hours. The condensation reaction of benzaldehyde with acetaldehyde was carried out with five-time excess of acetaldehyde to provide favorable equilibrium for the production of cinnamaldehyde. The kinetic analysis of this reaction shows similar results to the hydrolysis of cinnamaldehyde with respect to the temperature, NH_3_ concentration, and product yield. The reported work discovers a new area of base-catalyzed reactions in NCW however; the authors made simplifying assumptions that could impact the accuracy of the results. The gas phase ammonia concentration was calculated with the ideal gas law; which does not account for non-idealities resulting from high pressures in NCW.

The *tert*-butoxycarbonyl (*t*-Boc) group is commonly used to protect reactive amine groups in organic synthesis and peptide chemistry (ref). The deprotection of the *N*-Boc group is conventionally achieved by using strong acids such as HNO_3_ [73] and H_2_SO_4_ [74] and Lewis acids such as ZnBr_2_ [75,76]. However, NCW provides a benign and acid/base free method to carry out *t*-Boc deprotection reactions. G. Wang *et al.* [77] reported the *N*-Boc deprotection of various aromatic and aliphatic amines. The effect of temperature on the deprotection of *N*-Boc-aniline is shown in Table 9. The reaction proceeds slowly at 130 °C with 12% yield after two hours and 39% yield after four hours; 86% yield of *N*-Boc-aniline deprotection was observed at 150 °C after four hours. In the absence of water, the deprotection of *N*-Boc-aniline was not observed after four hours at 150 °C. The yield and reaction time for the *N*-Boc deprotection of aromatic compounds with electron donating substituents such as methyl (88% yield after 4 h), methoxy (96% yield after 4 hrs), and hydroxyl (95% after 2 h) groups were similar or better than that of *N*-Boc-aniline. The presence of electron withdrawing groups such as chlorine and nitro groups on the aromatic ring required longer times to achieve good yields; it took six hours for the chloro-substituted *N*-Boc-aniline to attain 87% yield and ten hours for the nitro-substituted *N*-Boc-aniline to reach 97% yield. Additionally, the deprotection of *N*-Boc-L-alanine showed complete conversion within two hours; it is suggested that the increased concentration of H^+^ ions resulting from the dissociation of the hydroxyl group in the *N*-Boc-L-alanine improved the acid-catalyzed reaction.

**Table 9:** Yield of water mediated *N*-Boc-aniline deprotection at different temperatures [77].

Entry	Temperature (°C)	Time (h)	Isolated yield (%)
1	30	4	0
2	80	4	0
3	130	2	12
4	130	4	39
5	150	2	38
6	150	4	86

**Scheme 10:**
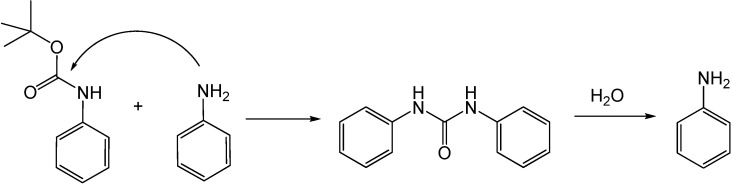
*N*-Boc-deprotection of aniline in NCW with diphenylurea as an intermediate.

Wang *et al.* [78] reported the *N*-Boc deprotection of aromatic heterocycles, aromatic amines, and aliphatic amines and amides in boiling water. The deprotection of *N*-Boc heterocycles such as imidazole, pyrazole, benzimidazole, and benzotriazole were achieved with quantitative yields in ten minutes at 100 °C. At the same conditions, the deprotection of *N*-Boc-indole took 4 hours, but electron-deficient analogs took shorter time, e.g., the complete deprotection of *N*-Boc-azaindole took 1 hour. The *N*-Boc deprotection of various aromatic molecules was also reported. The presence of substituents with hydrogen bonding ability on the aromatic ring - such as hydroxy, methoxy, and nitro – facilitated faster reactions due to their greater solubility in water. For instance, the deprotection of *N*-Boc-aniline required 10 hours of reaction time at 100 °C while the deprotection of *p*-nitroaniline was achieved in 3.5 hours at the same temperature. In addition, we are studying the effect of temperature on the deprotection of *N*-Boc-aniline in NCW [79,80]. Quantitative yields are obtained after 40 minutes at 150 °C, after 20 minutes at 200 °C, and after 10 minutes at 250 °C. Also, we identified diphenylurea as an intermediate in the reaction; we believe that aniline is acting as a nucleophile and it is reacting with *N*-Boc-aniline to form the intermediate, as shown in Scheme 10. 

**Figure 18:**
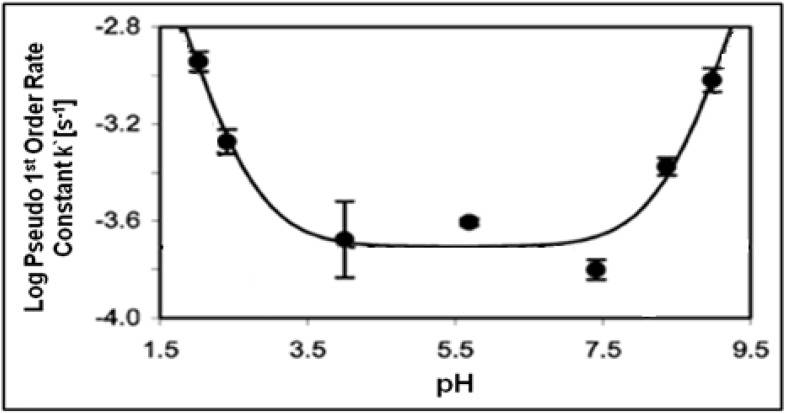
Pseudo-First-Order Rate Constant (k`) for bisphenol A cleavage to form *p*- isopropenylphenol as a function of system pH at 250 °C [82].

The examples of acid/base catalyzed reactions discussed above take advantage of the unique properties of NCW. It is important to understand the effects of changing the pH and to examine the underlying mechanism of the reaction and the effect of pH. Hunter *et al.* [81,82] reported the effect of changing the pH on the cleavage of bisphenol A to form *p*-isopropenylphenol. The pH was varied by adding H_2_SO_4_, HCl or NaOH to adjust the pH of water at 250 °C (pH = 5.7); the pseudo-first-order rate constant (k`) was modeled as a function of system pH is shown in Figure 18. 

Log(k`) has reaches maximum values of -2.94 at pH = 2.0 and a similar value of -3.02 at pH = 9.0. In the pH range of 3.5-7.5, the rate constant maintains minimum values with log(k`) of about -3.7. The results indicate that the bisphenol A cleavage is due to specific acid catalysis at low pH and specific base catalysis at high pH in NCW. The authors modeled the pseudo-first-order rate constant with the following equation:

k’=k_A_[H_3_O^+^] + k_B_[OH^-^] + k_W_

where k_A_ is the second-order rate constants for specific acid catalysis, k_B_ is second-order rate constants for specific base catalysis, and k_W_ is the pseudo-first-order rate constant resulting from general acid or general base catalysis due to water. The reported values are k_A_ = 0.094 ± 0.053 L/mol s, k_B_ = 0.19 ± 0.12 L/mol s, and k_W_= 2.0 × 10^-4^ ± 6.8 × 10^-5^ s^-1^. The high value of k_W_ indicates that neat NCW performs general acid or general base catalysis due to water; the relatively higher value of k_B_ compared to k_A_ was concluded to reveal general base catalysis as dominant under neutral pH conditions. 

## 4. Conclusions

The thrust of this presentation has been to achieve the greatest asset of heterogeneous catalysis—ease of separation and reuse—for homogeneous reactions, which bring a host of additional advantages, in lack of mass transfer limitations, in augmented rate, and above all in the very much enhanced selectivity. Tunable solvents such as OATS and nearcritical water (NCW) are solvent systems that allow the manipulation of phase behavior, as means to combine homogeneous reactions with heterogeneous separations. These solvent systems do allow increased reaction rates, improved selectivities, and facile heterogeneous separations. 

Rhodium-catalyzed hydroformylation of 1-octene and *p*-methylstyrene to produce nonanal and 2-*p*-tolylpropanal were investigated in THF/H_2_O and ACN/H_2_O. When compared to the conventionally run processes (heterogeneous liquid-liquid or liquid-solid), OATS-mediated hydroformylation of 1-octene showed (1) at least an order of magnitude improved rate, (2) efficient product recovery (99% of products recovered at 3 MPa of CO_2_ pressure) and (3) separation of the catalyst for subsequent recycles (up to five times without significant loss of activity). OATS were also used for the *Candida Antarctica* lipase B (CAL B) kinetic resolution of *rac*-1-phenylethyl acetate to *(R)-*1-phenylethanol with more than 99% enantiomeric excess. In addition, the hydrolysis of 2-phenylethyl acetate to 2-phenylethanol (2PE) in OATS was investigated. The enzyme was recycled six times and maintained 96% activity. Polyethylene glycol 400 (PEG)-organic tunable solvents have been reported for the palladium-catalyzed C-O couplings. High conversion (80%) and product selectivity (71%) were reported for the coupling of 1-bromo-3,5-dimethylbenzene and *o*-cresol with potassium hydroxide to produce *o*-tolyl-3,5-xylyl ether. 

The improved solubility of nonpolar organics and the increased dissociation constant of NCW facilitate homogeneous *in situ* acid/base catalysis without the need for added acid/base. Alkylation and acylation reactions were reported in NCW with satisfactory yields and without added acid/base. For example, without any added catalyst, the Friedel-Crafts alkylation of phenol with *tert*-butanol produced 2-*tert*-butylphenol with 10% molar yield and 4-*tert*-butylphenol with 20% molar yield after 50 hours at 275 °C. Hydrolysis of various benzoate esters in NCW showed autocatalytic behavior. Near complete base-catalyzed hydrolysis in NH_3_-enriched NCW of cinnamaldehyde with benzaldehyde yield of 50% are obtained after three hours at 240 °C and 52.8 mg/L NH_3_. The addition of NH_3_ improved the yield of benzaldehyde and increased the reaction rate by up to ten-times. 

Another example of NCW-mediated reactions is the deprotection of *t*-Boc groups from amine compounds. The yield of *N*-Boc-aniline deprotection is 86% in acid/base free water at 150 °C after four hours. Finally, we discuss the pH dependence of the cleavage of bisphenol A to form *p*- isopropenylphenol. The results indicate that the bisphenol A cleavage is due to specific acid catalysis at low pH and specific base catalysis at high pH in NCW.
